# Does local lavage influence functional recovery during lumber discectomy of disc herniation? One year's systematic follow-up of 410 patients

**DOI:** 10.1097/MD.0000000000005022

**Published:** 2016-10-21

**Authors:** Ru-Sen Zhu, Yi-Ming Ren, Jian-Jun Yuan, Zi-Jian Cui, Jun Wan, Bao-You Fan, Wei Lin, Xian-Hu Zhou, Xue-Li Zhang

**Affiliations:** a Department of Spine Surgery, Tianjin Union Medical Center, Tianjin, PR China; b Department of Orthopedics, Tianjin Medical University General Hospital, Tianjin, PR China.

**Keywords:** local lavage, low back pain, lumber disc herniation, microdiscectomy, sciatica

## Abstract

Lumbar disc herniation (LDH) is a common disease and lumbar discectomy is the most common surgical procedure carried out for patients with low back pain and leg symptoms. Although most researchers are focusing on the surgical techniques during operation, the aim of this study is to evaluate the effect of local intervertebral lavage during microdiscectomy.

In this retrospective study, 410 patients were operated on by microdiscectomy for LDH during 2011 to 2014. Retrospectively, 213 of them (group A) accepted local intervertebral irrigation with saline water before wound closure and 197 patients (group B) only had their operative field irrigated with saline water. Systematic records of visual analog scores (VAS), Oswestry disability Index (ODI) questionnaire scale scores, use of analgesia, and hospital length of stay were done after hospitalization.

The majority (80.49%) of the cases were diagnosed with lumber herniation at the levels of L4/5 and L5/S1. Fifty-one patients had herniations at 2 levels. There were significant decreases of VAS scores and ODI in both groups between preoperation and postoperation of different time points. VAS scores decreased more in group A than group B at early stage of postoperation follow-up. However, there were no statistically significant differences between 2 groups in using analgesia, VAS and ODI up to 1 month of follow-up.

Microdiscectomy for LDH offers a marked improvement in back and radicular pain. Local irrigation of herniated lumber disc area could relief dick herniation-derived low back pain and leg radicular pain at early stage of post-operation. However, the pain relief of this intervention was not noticeable for a long period.

## 1 Introduction

Lumber disc herniation (LDH) is caused by damage of the discs and the soft jel inside them pushes through the wall of them and presses against the nerves or the spinal cord, causing a burning pain in legs and pain in the back.^[1]^ Operation involving removal of the portion of the intervertebral disc compressing the nerve root or spinal cord (or both) is needed when all kinds of conservative treatments could not get a satisfied result or the patients comply one of the series of symptoms as Cauda equine.^[2]^ Although most researchers are focusing on the selections and efficacy of surgical techniques during operation, such as open microdiscectomy, microendoscopic discectomy, and others, postoperative disc herniation-derived low back pain and leg radicular pain should be given enough attention.^[3]^ In addition, there is a growing body of researches to suggest that the pathology mechanism and autoimmunity mechanism of LDH play a vital role in explaining the disc herniation-derived low back pain and leg radicular pain, but their exact effects and the mechanisms involved remain obscure.^[4,5]^ Available efficacy data supported that the use of analgesics, nonsteroidal anti-inflammatory drugs and epidural steroid injections probably could relieve the associated pain and improve the quality of life without radically changing the midterm outcome.^[6]^ In our study, based on researches of mechanisms of LDH and injection therapy, we speculate that local irrigation of herniated lumber disc area may have a positive effect. To verify this, this study systematically evaluates the effect of local lavage during microdiscectomy by assessing some outcomes of indexes of 410 patients, and tries to find that whether the local intervertebral irrigation is an efficient way in reducing postoperative low back and leg radicular pain.

## 2 Materials and methods

Ethical approval or patient consent was not required since the intervention measures of the present study were not necessary or did not cause harm to patients during surgery.

### 2.1 Patient population

In this retrospective study, 410 patients were operated on by microdiscectomy for LDH, and results of patients were analyzed statistically from January 2011 to March 2014. Retrospectively, the patients were divided into 2 groups according to different irrigation areas. Two hundred thirteen of them accepted the local intervertebral irrigation before wound closure (group A), and 197 patients only had their operative field irrigated (group B).

The inclusion criteria were as follows: a simple LDH with lumbar spine radiographs, computed tomography (CT), and magnetic resonance image (MRI) corresponding to the clinical symptoms of lumbar radiculopathy; no improvement for 3 to 6 months after conservative treatments including medication, physical therapy, and injections; a surgical procedure performed by the same spine surgeon at a single institution; and follow-up of at least 1 year. The exclusion criteria of the present study were defined as hard disc herniation, foraminal and extraforaminal disc herniations, and spinal instability; a severe neurologic deficit or spinal instability that requiring fusion, and other pathologic conditions such as fractures, tumors, or infections; a LDH with lateral recess stenosis, lumbar spine stenosis, or lumbar spondylolisthesis; patients who are too sick to undergo surgery; first attack of LDH; and microdiscectomy as a revision surgery.

### 2.2 Surgical technique

Microdiscectomy at the appropriate level was performed according to a standardized institutional surgical protocol. A 2- to 3 cm posterior midline skin incision was made over the appropriate disc space. By using a periosteal elevator, the paraspinal muscles were split and a Caspar retractor was placed. All procedures, including partial laminectomy, foraminotomy, and removal of the ligamentum flavum, were performed using the operating microscopic guidance. A small annulotomy was used, and the disc fragment was removed in the conventional manner under microscopic view. Extracted disc material was limited to the central disc pieces in the disc space and inner-annular fragments. The endplates were preserved without requiring curettage. After confirming adequate decompression and release of the nerve root by using the microprobe, the local lavage was applied. For group A, before wound closure, patients accepted the local intervertebral irrigation and nerve root canal irrigation with saline water carefully using the thin catheter (10∼12 mm). For group B, only the surgical field was copiously irrigated with saline water and each layer of the wound was closed after meticulous hemostasis. A total of 200 mL of 0.9% normal saline was injected in either group A or group B.

### 2.3 Clinical evaluation and follow-up

The patients completed a questionnaire consisting of a 10-point visual analog scale (VAS) (0–10, with 0 reflecting no pain) for low back and leg pain preoperatively and at each follow-up visit. Functional outcomes were scored preoperatively and at each follow-up visit according to the Oswestry Disability Index (ODI; 0%–100%). Additionally, using of analgesia, hospital length of stay, complications of surgery, and recurrence rate were also evaluated to assess the outcomes of the procedures. Office follow-ups were conducted 1 day, 1 week, 1 month, 6 months, and 12 months after the operation.

### 2.4 Statistical analysis

The statistical analysis was performed with the use of SPSS version 22.0 (SPSS Inc, Chicago, IL). The results of descriptive data analysis are shown as means ± standard deviations for continuous variables, and as frequencies and percentages for categorical variables. Continuous variables were analyzed using the student *t* test. The Mann–Whitney *U* tests and the *χ*^2^ test were used to compare variables between 2 groups with non-normal distributions, with averages expressed as medians and interquartile range. For the categorical variables, Fischer exact tests were performed between 2 independent groups. All *P* values ≤0.05 were considered statistically significant.

## 3 Results

### 3.1 Baseline characteristics

**Figure 1:**
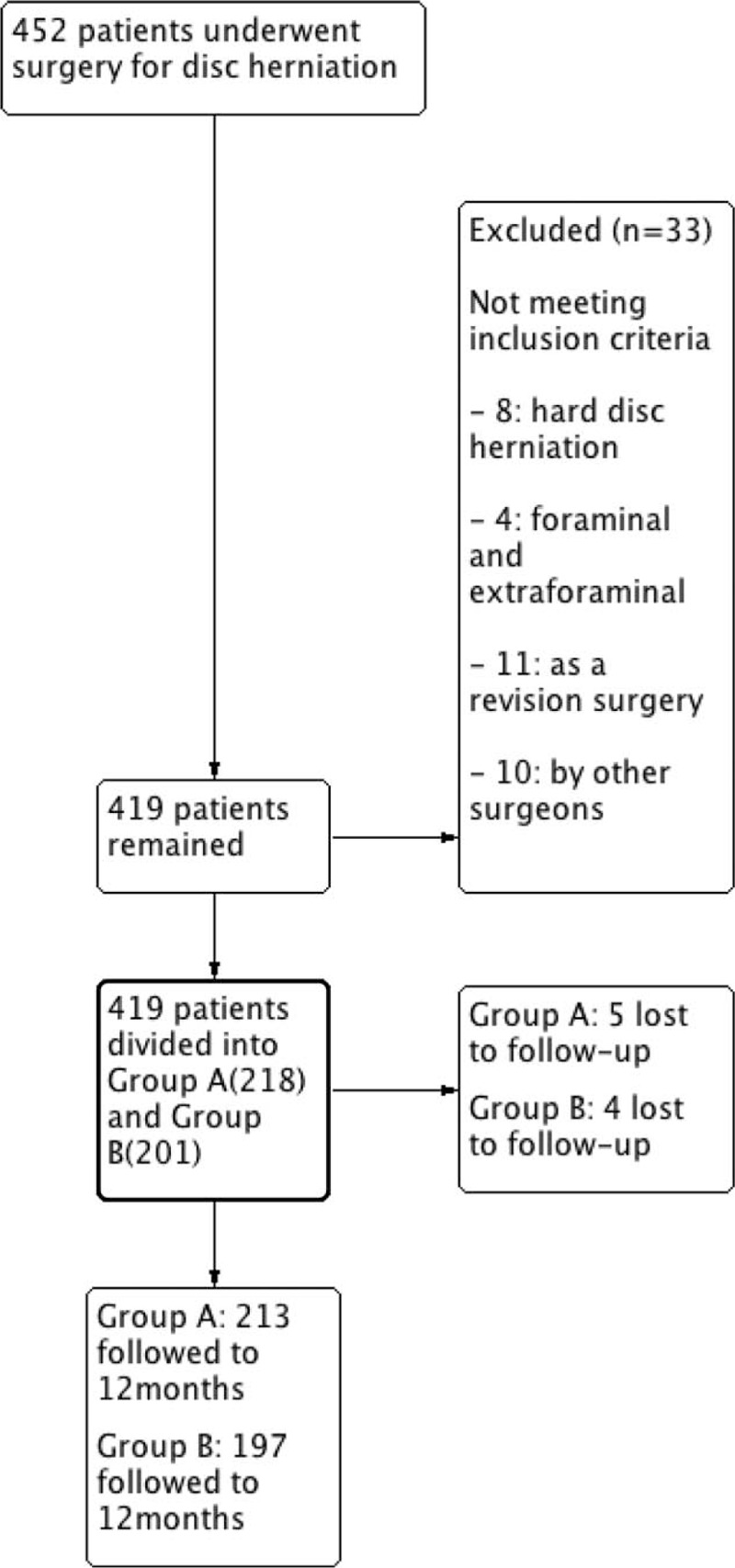
Flow diagram. Patient enrollment and follow-up.

A total of 452 patients were recruited to the study. Eight patients with hard disc herniation, 4 with foraminal and oraminal disc herniations, 11 treated by revision surgery, and 10 treated by other surgeons who were not meeting inclusion criteria were excluded. Nine were lost to follow-up: 5 from the group A and 4 from the group B. Finally, 213 patients in group A and 197 patients in group B met the inclusion and exclusion criteria (Fig. 1). The demographic results of the patients are summarized in Table 1. There were 226 men and 184 women with a mean age of 48.00 years (range, 16–82 years) included in the study. The majority (80.49%) of the cases were diagnosed with lumber herniation at the levels of L4/5 and L5/S1. Fifty-one patients had herniations at 2 levels. The average lengths of hospital stay for the group A and group B were 15.32 and 15.65 days respectively, including the time of short-term postoperative rehabilitation. Both groups followed to nearly 1 year.

**Table 1:**
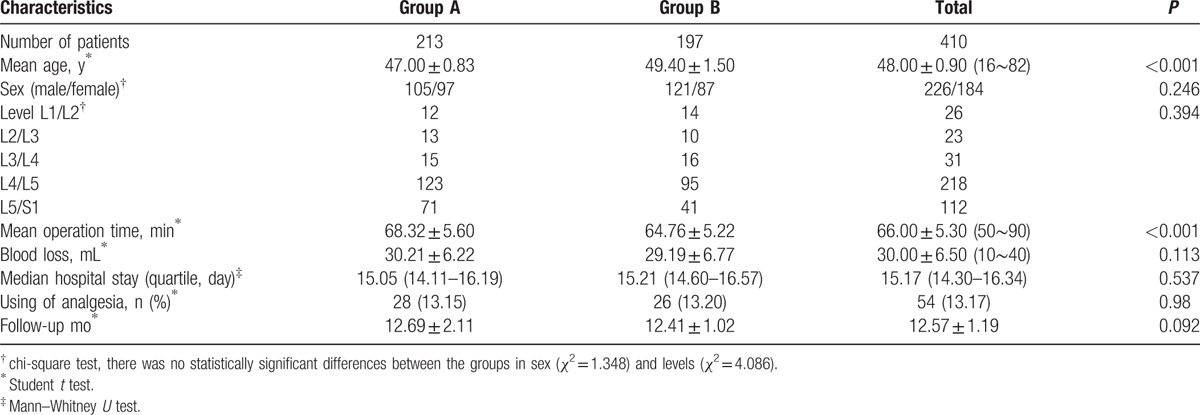
Demographic data.

### 3.2 Clinical outcomes

There were significant decreases of VAS scores and ODI in both groups between preoperation and postoperation of different time points in Table 2. VAS back scores decreased more in group A (2.66 ± 0.71) than group B (3.52 ± 0.70) with significance (*P* < 0.001) at follow-up of 1 day. Similarly, VAS back scores were significantly shorter in group A (2.51 ± 0.60) as compared with group B (2.67 ± 0.76) (*P* = 0.018) at follow-up of 1 week. VAS leg scores in group A decreased more than in group B at early stage of postoperation follow-up (1 day and 1 week), but there were no statistically significant differences (*P* > 0.05). In addition, ODI decreased more in group A (12.16 ± 2.57) than in group B (13.89 ± 1.77) with significance (*P* < 0.001) at follow-up of 1 day. However, there were no statistically significant differences between 2 groups in VAS and ODI up to 1 month of follow-up.

**Table 2:**
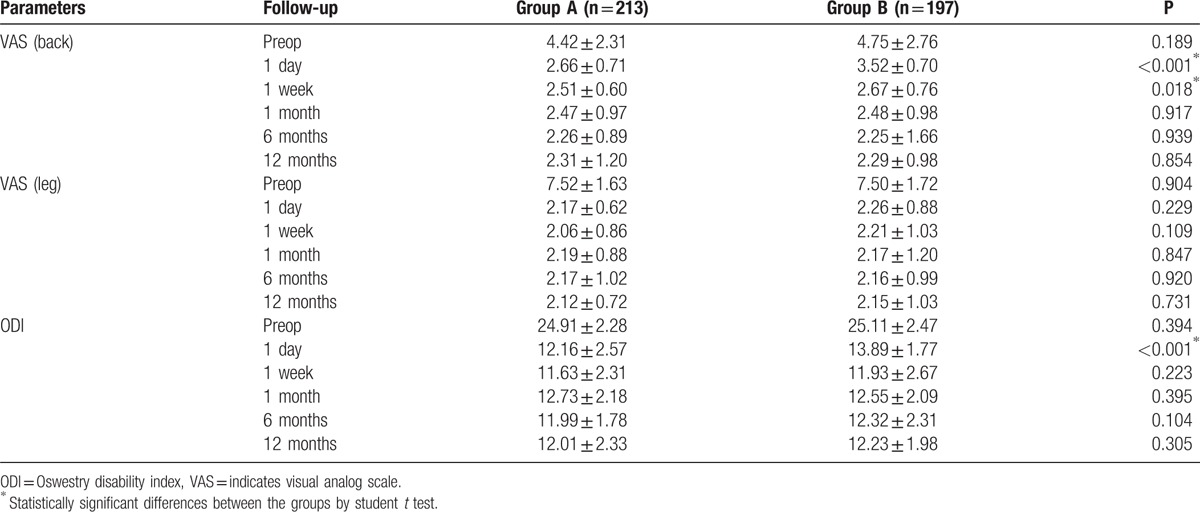
Clinical outcomes according to the parameters.

Some perioperative outcomes were shown in Table 1. The mean operative time for group A was 68.32 minutes, which was slightly longer than the 64.76 minutes of mean operative time for group B (*P* < 0.001). Both groups had negligible blood loss with no clinical significance (*P* > 0.05). There were no statistically significant differences between group A (28 [13.15%]) and group B (26 [13.20%]) in using of analgesia at early stage of postoperation follow-up.

### 3.3 Complications and recurrences

In Table 3, complications occurred in 6 patients (2.8%) in group A and 8 patients (4.1%) in group B. Only 1 patient with postoperative infection was reported. None of temporary nerve root injury and discitis was found after surgery. Postoperative epidural hematoma occurred in 1 patient in group A and 2 patients in group B, which were successfully removed with evacuation. Postoperative cerebrospinal fluid (CSF) leakage owing to dural tear was reported for group A (1 [0.5%]) and group B (2 [1.0%]) approaches, which was successfully managed conservatively. It was covered with gelfoam intraoperatively, and the patients were given epidural steroid agents and needed bed rest for 48 hours. No delayed CSF fluid leaks or pseudomeningoceles developed. Re-herniation at 12 months occurred in 4 patients in group A (1.9%) and 4 patients in group B (2.0%), which showed no statistically significant difference (*P* < 0.01). The patients in both groups with re-herniation underwent re-operation with open lumbar discectomy.

**Table 3:**
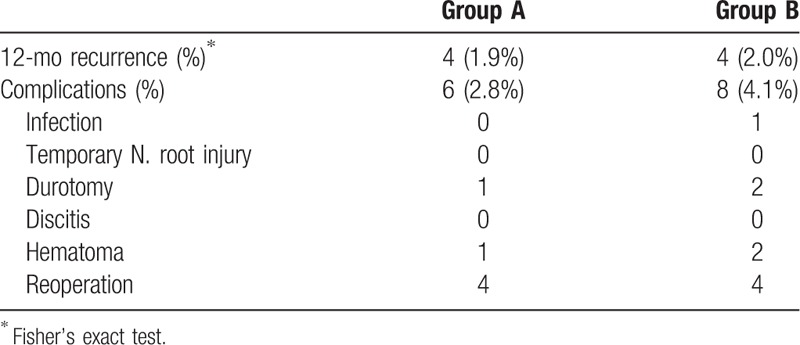
Summary of complications and recurrence rate.

## 4 Discussion

Conventional microdiscectomy remains the criterion standard for treating a herniated lumbar intervertebral disc and has been established as an alternative to traditional, more aggressive open approaches for the treatment of LDH. With limited blood loss, shorter duration of surgery, faster postoperative recovery, and smaller incision which is used to insert surgical instruments under x-ray guidance, our results proved once again that this surgery can more extensively preserve normal paraspinal structures and effectively address a herniated disc in the lumbar, spine which is causing low back and leg pain.^[7,8]^

More and more researches suggested that the pathology mechanism and autoimmunity mechanism of LDH play an important role in explaining the disc herniation-derived low back pain and leg radicular pain.^[9–16]^ Intraoperative epidural steroids are effective in reducing low back or leg radicular pain in the early stage and reducing consumption of analgesia, which might be associated with autoimmunity in intervertebral disc herniation and function of inflammatory cytokines.^[17–19]^ However, with this widespread but nonstandardized use of epidural steroids after lumbar discectomy, a better understanding of their associated risks such as a symptomatic CSF leak and the infection, is warranted.^[20–21]^ Local intervertebral irrigation with saline water, which has a better safety, is recommended as the optical alternative for epidural steroid injections. In the present study, VAS scores and ODI decreased significantly in both groups between preoperation and post-operation of different time points. The VAS scores of patients of group A who accepted the local intervertebral irrigation and nerve root canal irrigation decreased more than that of patients of group B who only accepted local irrigation copiously in the surgical field, and group A's ODI was less at short-term follow-up. In addition, there were no statistically significant differences of VAS and ODI between 2 groups at long-term follow-up. Taken together, we hold that local irrigation mainly decreased the early postoperative pain of patients and low back pain decreased more significantly. In Scuderi et al's study,^[22]^ they performed epidural space lavage followed by a highly sensitive protein assay on a group of patients with symptomatic disc herniation or spinal stenosis with radiculopathy and aim to identify specific inflammatory mediators in the epidural space, which participate in the degenerative cascade. In another study, researchers found that a molecular complex of fibronectin and aggrecan from the epidural lavage predicts response to lumbar epidural steroid injection for radiculopathy with herniated nucleus pulposus.^[23]^ To sum up, we hypothesize that the local intervertebral and nerve root canal irrigation may be able to change the release of inflammatory cytokines and trigger the autoimmunity in irrigated intervertebral disc and nerve root canal. On one hand, inflammatory factors and small disc fragments generated during surgery that continuously stimulated and caused related pain can be washed away by local lavage. On the other hand, only 1 patient with postoperative infection was reported in group B. In addition, intraoperative local lavage is deemed to be helpful for the clean of the operation field by surgeons, and could remove inflammatory factors, dilute or remove aseptic inflammatory factors and bacterial colonies. However, further studies still will be needed to confirm whether local lavage can help prevent an infection.^[24]^ Taken together, we deem that the local lavage actually does well to the functional recovery after lumber discectomy of disc herniation.

No serious adverse effects were encountered with the procedure in the present study. The rates of recurrent disc herniation in the 2 groups were 1.9% (4/213) and 2.0% (4/197), respectively. The rates correspond to the data in the previous literature and possible reasons are listed as followed. First, the operation resulted in the destruction of the ligament and annulus fibrous in the lesion area, and the postoperative residual intervertebral disc tissues escaped from the annular defects, leading to a reherniation.^[25]^ Second, a significantly higher recurrence rate was related with larger anular defects and less disc removal. The more aggressive removal of remaining intervertebral disc material may decrease the risk of re-herniation.^[26]^

It is important to note that the present study has several limitations. First, this study is retrospective, which may have complicated our outcome analysis. In addition, selection bias and information bias are easy to produce and hard to avoid. In the future, a prospective controlled study should be conducted to provide more detailed information with larger sample and longer follow-up. Second, operation time, blood loss, and other clinical heterogeneity were not discussed specifically, causing that the same surgery method was adopted and the same group of doctors performed the surgery. Whether these factors can affect the outcomes of treatment is unknown. Third, the results of the present study should be regarded as only preliminary. Many issues remain unresolved, such as the actual mechanism of action, the duration of outcomes, and long-term consequences. Further studies are needed to clarify this problem.

## 5 Conclusion

Our study showed that microdiscectomy for LDH offers a marked improvement in back and radicular pain, and local irrigation with saline water of herniated lumber disc area could relief dick herniation-derived low back pain and leg radicular pain at early stage of postoperation. However, the pain relief of this intervention was not noticeable for a long period.
